# Regulation mechanisms of disulfidptosis-related genes in ankylosing spondylitis and inflammatory bowel disease

**DOI:** 10.3389/fimmu.2024.1326354

**Published:** 2024-02-16

**Authors:** Lin Li, Haixin Fang, Fuzhen Li, Kunpeng Xie, Pengyi Zhou, Haiyan Zhu, Xuemin Jin, Ruifeng Song, Peizeng Yang, Du Liping

**Affiliations:** ^1^ Department of Ophthalmology, Henan International Joint Research Laboratory for Ocular Immunology and Retinal Injury Repair, The First Affiliated Hospital of Zhengzhou University, Henan Province Eye Hospital, Zhengzhou, Henan, China; ^2^ The Academy of Medical Sciences, Zhengzhou University, Zhengzhou, China; ^3^ Department of Gastroenterology, The First Affiliated Hospital of Zhengzhou University, Zhengzhou, Henan, China; ^4^ Chongqing Key Laboratory of Ophthalmology and Chongqing Eye Institute, The First Affiliated Hospital of Chongqing Medical University, Chongqing, China

**Keywords:** bioinformatics, mendelian randomization, disulfidptosis, ankylosing spondylitis, ulcerative colitis, Crohn’s disease

## Abstract

**Introduction:**

Disulfidptosis is a recently identified form of cell death that contributes to maintaining the internal environment balance of an organism. However, the molecular basis of disulfidptosis in ulcerative colitis (UC), ankylosing spondylitis (AS), and Crohn’s disease (CD) has not been thoroughly explored.

**Methods:**

Firstly, the differentially expressed genes (DEGs) and disulfidptosis-associated genes (DAGs) were obtained through differential analysis between diseases (AS, CD, and UC) and control groups. After the disulfidptosis score was acquired using the single-sample gene set enrichment analysis (ssGSEA) algorithm, the DE-DAGs were screened by overlapping DAGs and DEGs of the three diseases. Next, the feature genes were selected through a combination of machine learning algorithms, receiver operating characteristic (ROC) curves, and expression analysis. Based on these feature genes, nomograms were created for AS, CD and UC. The co-feature genes were then identified by taking the intersections of the genes featured in all three diseases. Meanwhile, single-gene set enrichment analysis (GSEA) and the TF-mRNA-miRNA network were utilized to investigate the molecular mechanisms of the co-feature genes. To validate the expression differences of the co-feature genes between healthy controls and patients (AS and IBD), RT-PCR was performed. Lastly, mendelian randomization (MR) analysis was utilized to explore the causality between genetic variants of *S100A12* with AS, UC and CD.

**Results:**

In this study, 11 DE-DAGs were obtained. Functional enrichment analysis revealed their involvement in cytokine production and fatty acid biosynthesis. Latterly, AS/CD/UC -feature genes were derived, and they all had decent diagnostic performance. Through evaluation, the performance of the nomogram was decent for three diseases. Then, 2 co-feature genes (*S100A12* and *LILRA5*) were obtained. The GSEA enrichment results indicated that the co-feature genes were mainly enriched in the cytokine-cytokine receptor interaction and drug metabolism cytochrome P450. As shown by functional experiments, there was a correlation between the mRNA expression of *S100A12* with AS, UC and CD. Additionally, a causal connection between *S100A12* and IBD was detected through MR analysis.

**Discussion:**

In this study, 2 co-feature genes (*S100A12* and *LILRA5*) were screened, and their functions were investigated in AS, CD and UC, providing a basis for further research into diagnosis and treatment.

## 1 Introduction

Ankylosing spondylitis (AS) and inflammatory bowel disease (IBD) are two common autoimmune conditions, usually share common clinical features such as sacroiliitis, dactylitis and enthesitis, as well as extra-articular manifestations such as intestinal inflammation, psoriasis and uveitis (1, 2). AS is a prevalent chronic immune-mediated condition characterized by inflammation in the axial skeleton. Epidemiological research indicates that AS has a worldwide incidence rate ranging from 0.1% to 1.4%, with a higher occurrence in males compared to females (3). IBD is a chronic condition impacting the digestive system and can manifest in various forms, with the most prevalent subtypes being ulcerative colitis (UC) and Crohn’s disease (CD) (4). Over the past decade, the incidence of IBD has increased from 0.3% to 1.3% and continues to rise (5). Common symptoms in IBD patients include weight loss, diarrhea, rectal bleeding and abdominal pain (6). Additionally, IBD patients frequently experience extraintestinal manifestations (EIMs) involving various organs, including ocular, cutaneous, hepatic, biliary, and hematologic complications (7). Arthritis is the most prevalent EIM, affecting up to 40% of IBD patients, with a higher occurrence in CD compared to UC (8).

The coexistence of AS and IBD is prevalent, with research indicating that the incidence of IBD in AS patients ranges from 6% to 14%, and the risk of developing AS in IBD patients is approximately 3.7% to 4.5% (9, 10). A study conducted in Germany reported that 5% to 10% of AS cases are linked to IBD, and a greater proportion of AS patients displayed subclinical intestinal inflammation (11). AS patients bear a persistent risk of developing IBD throughout their lives, and this risk rises as the disease duration prolongs (12). Both AS and IBD are chronic recurrent conditions that significantly impact the quality of life for affected individuals (13, 14).

Cell death is a physiological process essential for the regulation of biological development and internal environmental stability. It encompasses various mechanisms, including apoptosis, necroptosis, pyroptosis, ferroptosis, NETosis, as well as cell death processes associated with autophagy and non-programmed necrosis (15–17). Recent researches have indicated a connection between IBD and AS with various types of cell death (18, 19). However, there has been limited exploration of the relationship between various forms of cell death and the occurrence of these two diseases. According to a recent study, it was found that cells expressing elevated *SLC7A11* levels can prevent ferroptosis when glucose is scarce by absorbing cystine through *SLC7A11*-mediated mechanisms. However, this process may potentially result in a distinct form of cell death known as disulfidptosis (20).

To gain a more profound insight into the regulatory mechanisms of the genes related to disulfidptosis in patients with AS and IBD. This study applies bioinformatics techniques and experimental validation to investigate the shared regulatory mechanisms of disulfidptosis-related genes in both of these conditions, offering new targets and prospects for the treatment of the diseases.

## 2 Materials and methods

### 2.1 Data source

The GSE25101, GSE75214, GSE73754, GSE102133 and GSE16879 datasets were sourced from the GEO database (**Supplementary Data 1**). The GSE25101 dataset (GPL6947) includes the RNA-seq data of whole blood from 16 control samples and 16 AS samples. The GSE75214 dataset (GPL6244) includes the RNA-seq data of intestinal mucosal biopsies tissue from 11 control samples and 51 CD samples. The GSE75214 dataset (GPL6244) also includes the RNA-seq data of intestinal mucosal biopsies tissue from 11 control samples and 74 UC samples. The GSE73754 dataset (GPL10558) includes whole blood samples from 52 AS and 20 control samples. The GSE102133 dataset (GPL6244) includes the RNA-seq data of ileal mucosa tissue from 65 CD samples and 12 control samples. The GSE16879 dataset (GPL570) includes the RNA-seq data of colonic mucosal biopsy tissue from 24 UC samples and 6 control samples. Of these, the GSE25101 and GSE75214 datasets were utilized as the training cohorts and the GSE73754, GSE102133 and GSE16879 datasets were utilized as the validation cohorts. Then, 4 disulfidptosis genes (*SLC7A11*, *SLC3A2*, *RPN1* and *NCKAP1*) were obtained from previous report (20). The available GWAS summary statistics correlated with AS, UC and CD was derived from the GWAS meta-analysis of The Genotype-Tissue Expression (GTEx) Consortium (gtexportal.org).

### 2.2 Identification of differentially expressed genes

DEGs1 (AS vs control), DEGs2 (CD vs control) and DEGs3 (UC vs control) were selected in two training cohorts by the limma package (v 3.54.0) (21) with P value < 0.05 and |log_2_FC| > 0.25, respectively. The results of the differential analysis were visually represented using volcano maps and heatmaps. The volcano maps and heatmaps were generated by the ggplot2 package (v 3.4.1) (22) and pheatmap package (v 1.0.12), respectively.

### 2.3 Identification and functional enrichment of differentially expressed disulfidptosis-associated genes

The GSVA package (v 1.46.0) (23) was utilized to estimate disulfidptosis scores for each of the three disease samples using the ssGSEA algorithm, and the patient samples were categorized into high and low-score groups with the median score serving as the threshold for classification. DAGs1 (AS-high-disulfidptosis scores vs AS-low-disulfidptosis scores), DAGs2 (CD-high-disulfidptosis scores vs CD-low-disulfidptosis scores) and DAGs3 (UC-high-disulfidptosis scores vs UC-low-disulfidptosis scores) were selected using the limma package (v 3.54.0) (21) with P value < 0.05 and |log_2_FC| > 0.25, respectively. The results of differential analysis were depicted using both volcano plots and heatmaps. The intersection of DAGs1 and DEGs1, DAGs2 and DEGs2, and DAGs3 and DEGs3 was taken to obtain DE-DAGs1, DE-DAGs2 and DE-DAGs3 respectively. The DE-DAGs were filtered by overlapping DE-DAGs1, DE-DAGs2 and DE-DAGs3. Gene Ontology (GO) and Kyoto Encyclopedia of Genes and Genomes (KEGG) enrichment analyses of DE-DAGs were implemented by the clusterProfiler package (v 4.7.1) (24). Enrichment analysis outcomes were regarded as statistically significant if the adjusted p-value was less than 0.05.

### 2.4 Machine learning screening of candidate feature genes

In our study, XGBoost analysis was conducted on the basis of DE-DAGs to acquire XGBoost-feature genes1 (AS), XGBoost-feature genes2 (CD) and XGBoost-feature genes3 (UC) by glmnet package (v 4.1-4). In the meantime, Random Forest (RF) algorithm was created out on the basis of DE-DAGs to acquire RF-feature genes1 (AS), RF-feature genes2 (CD) and RF-feature genes3 (UC). The Gini coefficient of DE-DAGs was computed using the RF algorithm, and the genes before the significant decrease in the Gini coefficient were selected as RF-feature genes. Lastly, SVM-RFE analysis was created out on the basis of DE-DAGs to acquire SVM-RFE-feature genes1 (AS), SVM-RFE-feature genes2 (CD) and SVM-RFE-feature genes3 (UC), respectively. Then, the AS-candidate-feature genes were filtered via overlapping XGBoost-feature genes1, RF-feature genes1 and SVM-RFE-feature genes1; the CD-candidate-feature genes were filtered via overlapping XGBoost-feature genes2, RF-feature genes2 and SVM-RFE-feature genes2; the UC-candidate-feature genes were filtered via overlapping XGBoost-feature genes3, RF-feature genes3 and SVM-RFE-feature genes3.

### 2.5 Selection and verification of feature genes

To explore the ability of candidate feature genes to distinguish between control and disease (AS, CD and UC) groups, their expression levels between disease and control groups were compared and receiver operating characteristic (ROC) curves were plotted for these genes in both the training and the validation cohort. The expression analysis results were presented by box plots. The area under the curve (AUC) values of ROC curves were computed using the pROC package (v1.18.0) (25). The genes with consistent expression trends and significant differences between groups with AUC values exceeding 0.7 in both the training and validation cohorts were screened as feature genes (AS, CD, and UC) for subsequent analysis.

### 2.6 Construction of the nomogram

To predict the risk of AS, CD, and UC, nomograms were constructed based on feature genes for AS, CD, and UC. Next, calibration curve and decision curve analysis (DCA) curves were plotted to judge the performance of the nomogram.

### 2.7 Single-gene set enrichment analysis analysis

To investigate the impact of the expression of co-feature genes on pathways in the three disease groups, single-gene GSEA analysis was performed. The co-feature genes were screened by overlapping AS-feature genes, CD-feature genes, and UC-feature genes. Then, the correlation of co-feature genes to all other genes in AS-, CD-, and UC-related datasets, and all the genes were sorted according to the correlation from high to low. The ranked genes were taken as the gene set to be tested, and the KEGG signaling pathway was taken as the pre-defined gene set to detect its enrichment in the gene set via clusterProfiler package (v 4.4.4) (24). The top 5 results for KEGG significance were visualized separately.

### 2.8 Screening for co-feature genes and immune-infiltration analysis

The proportion of 22 immune cell subtypes was computed separately for each sample by the CIBERSORT algorithm (26) in the GSE25101 and GSE75214 cohorts. The difference in the proportion of immune cells (filter out immune cells whose cell abundance was 0 in more than 75% of the samples) infiltrating between disease and control groups was compared using the Wilcoxon method. Meanwhile, the correlation analysis was performed between immune cells and co-feature genes.

### 2.9 Construction of TF-mRNA-miRNA network

To obtain the regulatory factors of co-feature genes, we carried out a TF-mRNA-miRNA network. In this study, miRNet database was employed to predict the miRNAs that potentially target the co-feature genes. The TFs of co-feature genes were retrieved using the NetworkAnalyst database. Lastly, the network was visualized using Cytoscape software (v3.9.0) (27).

### 2.10 Functional validation of the co-feature genes

To validate the expression of co-feature genes, 13 normal controls, 8 AS patients, 12 UC patients, and 2 CD patients were enrolled in this study. The 1984 New York Diagnostic Criteria served as the principle for diagnosing AS (28). The diagnosis of IBD with its 2 main sub-forms (CD and UC), is based on clinical, endoscopic, radiologic, and histologic criteria (29). Our study followed the ethical guidelines outlined in the Helsinki Declaration and received ethical approvals from the research committees of both the First Affiliated Hospital of Zhengzhou University (2021-KY-0246-001) and the First Affiliated Hospital of Chongqing Medical University (No. 2009-201008). Peripheral blood mononuclear cells (PBMCs) were collected from the participants’ blood and used for RNA extraction. RT-PCR were conducted to assess the mRNA expression of *S100A12* and *LILRA5*. The amplification system and primer sequences used for *beta-actin*, *S100A12* and *LILRA5* can be found in **Tables 1** and **2**, respectively. Gene expression levels were determined using the 2^-ΔΔCt^ method. GraphPad Prism 9.5 was employed for data visualization and graph creation.

**Table 1:** Amplification system of RT-PCR.

Step	Temperature	Time	Cycle
Step 1	95°C	30 sec	1
Step 2	95°C	5 sec	40
	60°C	30 sec	
Step 3	Dissociation		

**Table 2:** Primer sequences used for *beta-actin*, *S100A12* and *LILRA5*.

Gene	Primer
*Beta-actin*	Forward: 5′GGATGCAGAAGGAGATCACTG3′
	Reverse: 5′CGATCCACACGGAGTACT3′
*S100A12*	Forward: 5′TGAAGAGCATCTGGAGGGAAT3′
	Reverse: 5′GGTGTCAAAATGCCCCCTTCC3′
*LILRA5*	Forward: 5′AGCTGGTGGTGACAGGATTC3′
	Reverse: 5′AACCTGTCGAATCTCAGCCG3′

### 2.11 Mendelian randomization analyses

MR was employed to detect the causal relationship between co-feature gene expression levels and the diseases. Single-nucleotide polymorphisms (SNPs) were defined as instrumental variables (IVs). Gene data were sourced from publicly available Genome-Wide Association Study (GWAS) datasets. These data of outcomes were obtained from 10,619, 487,598, and 51,874 subjects of European population, respectively (ID: ebi-a-GCST005529, ebi-a-GCST90038684 and ieu-a-12). MR analysis was conducted using the ‘Two Sample MR’ package, and Inverse variance weighted (IVW) method was applied to assess the relationship between co-feature gene levels (cause) and the diseases (effect). Finally, additional sensitivity analysis was performed using MR-Egger.

## 3 Results

### 3.1 Screening of DEGs

**Figure 1:**
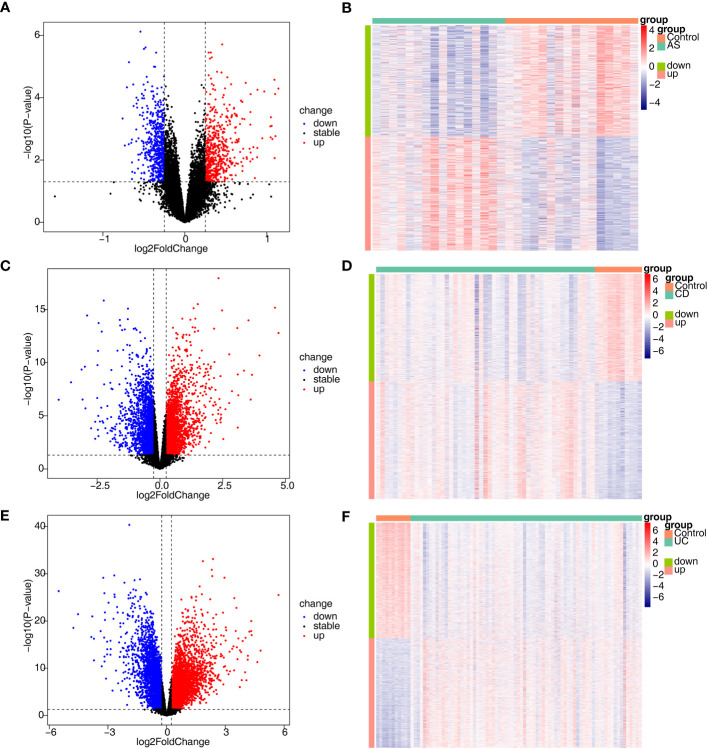
Identification of DEGs associated with AS, CD and UC. **(A, B)** The volcano plot and heatmap plot of DEGs identified in GSE25101 (AS, n=32, p < 0.05); **(C, D)** The volcano plot and heatmap plot of DEGs identified in GSE75214 (CD, n=62, p < 0.05); **(E, F)** The volcano plot and heatmap plot of DEGs identified in GSE75214 (UC, n=85, p < 0.05).

In total, 1134 DEGs1 (573 upregulated genes, 561 downregulated genes, **Supplementary Table 1**), 4629 DEGs2 (2421 upregulated genes, 2208 downregulated genes) and 7529 DEGs3 (3668 upregulated genes, 3861 downregulated genes) were acquired between disease and control samples (**Figures 1A–F**).

### 3.2 Functional annotation of DE-DAGs

**Figure 2:**
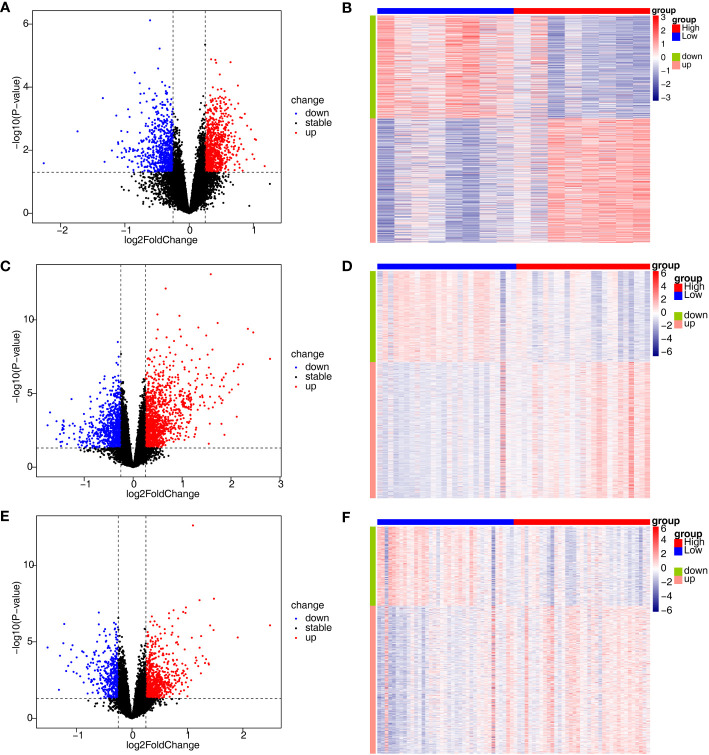
Identification of DEGs associated with disulfidptosis involved in AS, CD and UC. **(A, B)** The volcano plot and heatmap plot of DAGs involved in AS (p < 0.05); **(C, D)** The volcano plot and heatmap plot of DAGs involved in CD (p < 0.05); **(E, F)** The volcano plot and heatmap plot of DAGs involved in UC (p < 0.05).

**Figure 3:**
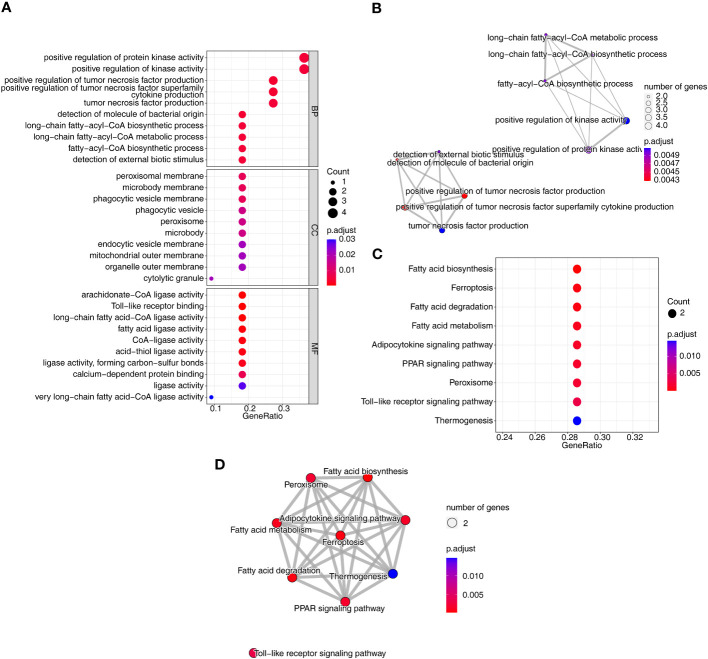
Identification and functional analysis of co-DE-DAGs associated with AS, CD and UC. **(A)** Bubble plot of GO functional enrichment; **(B)** Network Diagram of GO functional enrichment; **(C)** Bubble plot of KEGG functional enrichment; **(D)** Network Diagram of KEGG functional enrichment.

To investigate the relationship between the three diseases and disulfidptosis, the disulfidptosis score was computed and DAGs were obtained. A total of 1466 DAGs1 (802 upregulated genes, 664 downregulated genes), 2133 DAGs2 (855 upregulated genes, 1278 downregulated genes) and 1139 DAGs3 (742 upregulated genes, 397 downregulated genes) were gained (**Figures 2A–F**; **Supplementary Table 2**). After taking the intersections, 422 DE-DAGs1, 1496 DE-DAGs2 and 1064 DE-DAGs3 were obtained (**Supplementary Table 3**). Finally, 11 DE-DAGs (*S100A12*, *TLR1*, *SERPINB1*, *ACSL4*, *LY96*, *RRAGD*, *LILRA5*, *HECW2*, *ACSL1*, *ANXA3*, and *NRG1*) were screened (**Supplementary Table 4**). The results of the enrichment analysis revealed that the DE-DAGs were associated with 92 GO entries and 24 KEGG pathways. The GO term annotation showed that intersecting genes were primarily involved in processes such as fatty-acyl-CoA biosynthetic process, cytokine production, etc. (**Figures 3A, B**; **Supplementary Table 5**). KEGG enrichment results included ferroptosis, fatty acid biosynthesis, etc. (**Figures 3C, D**; **Supplementary Table 6**).

### 3.3 Screening for three disease candidate feature genes

**Figure 4:**
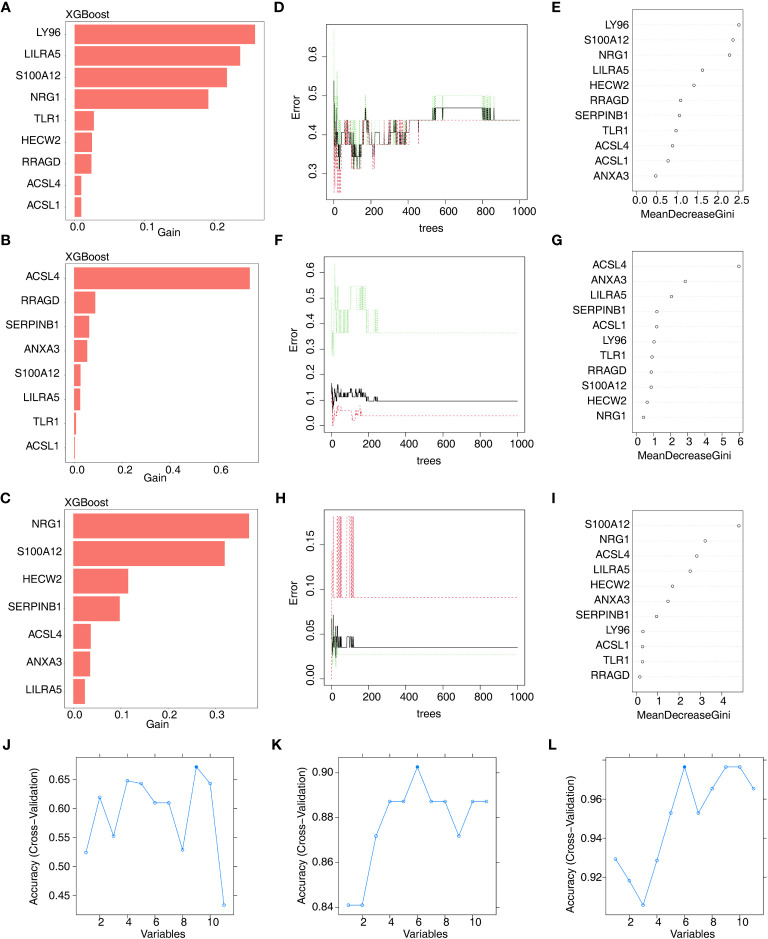
Machine learning screening of candidate feature genes involved in AS, CD and UC. **(A–C)** 9 XGBoost-feature genes1 (AS), 8 XGBoost-feature genes2 (CD) and 7 XGBoost-feature genes3 (UC) on the basis of DE-DAGs; **(D–I)** 5 RF-feature genes 1 (AS), 9 RF-feature genes 2 (CD) and 7 RF-feature genes 3 (UC) on the basis of DE-DAGs; **(J–L)** 9 SVM-RFE-feature genes1 (AS), 6 SVM-RFE-feature genes 2 (CD) and 6 SVM-RFE-feature genes 3 (UC) on the basis of DE-DAGs.

**Figure 5:**
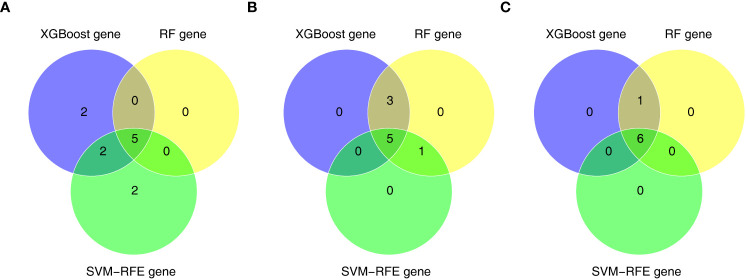
**(A)** 5 AS-candidate-feature genes, **(B)** 5 CD-candidate-feature genes and **(C)** 5 UC-candidate-feature genes were filtered via overlapping XGBoost-feature genes, RF-feature genes and SVM-RFE-feature genes.

Furthermore, we conducted gene screening using machine learning algorithms. In total, 9 XGBoost-feature genes1 (*LY96*, *LILRA5*, *S100A12*, *NRG1*, *TLR1*, *HECW2*, *RRAGD*, *ACSL4*, and *ACSL1*), 8 XGBoost-feature genes2 (*ACSL4*, *RRAGD*, *SERPINB1, ANXA3*, *S100A12*, *LILRA5*, *TLR1*, and *ACSL1*), 7 XGBoost-feature genes3 (*NRG1*, *S100A12*, *HECW2*, *SERPINB1*, *ACSL4*, *ANXA3*, and *LILRA5*) were mined (**Figures 4A–C**); 5 RF-feature genes1 (*LY96*, *S100A12*, *NRG1*, *LILRA5*, and *HECW2*), 9 RF-feature genes2 (*ACSL4*, *ANXA3*, *LILRA5*, *SERPINB1*, *ACSL1*, *LY96*, *TLR1*, *RRAGD*, and *S100A12*), 7 RF-feature genes3 (*S100A12*, *NRG1*, *ACSL4*, *LILRA5*, *HECW2*, *ANXA3*, and *SERPINB1*) were uncovered (**Figures 4D–I**); 9 SVM-RFE-feature genes1 (*S100A12*, *LY96*, *HECW2*, *SERPINB1*, *ACSL4*, *NRG1*, *ANXA3*, *LILRA5*, and *ACSL1*), 6 SVM-RFE-feature genes2 (*ACSL4*, *ANXA3*, *LILRA5*, *S100A12*, *ACSL1*, and *LY96*), 6 SVM-RFE-feature genes3 (*S100A12*, *ACSL4*, *NRG1*, *LILRA5*, *HECW2*, and *ANXA3*) were acquired after screening (**Figures 4J–L**). Hence, a total of 5 AS-candidate-feature genes (*LY96*, *LILRA5*, *S100A12*, *NRG1*, and *HECW2*), 5 CD-candidate-feature genes (*ACSL4*, *ANXA3*, *S100A12*, *LILRA5*, and *ACSL1*) and 6 UC-candidate-feature genes (*NRG1*, *S100A12*, *HECW2*, *ACSL4*, *ANXA3*, and *LILRA5*) were screened (**Figures 5A–C**).

### 3.4 Identification of feature genes

**Figure 6:**
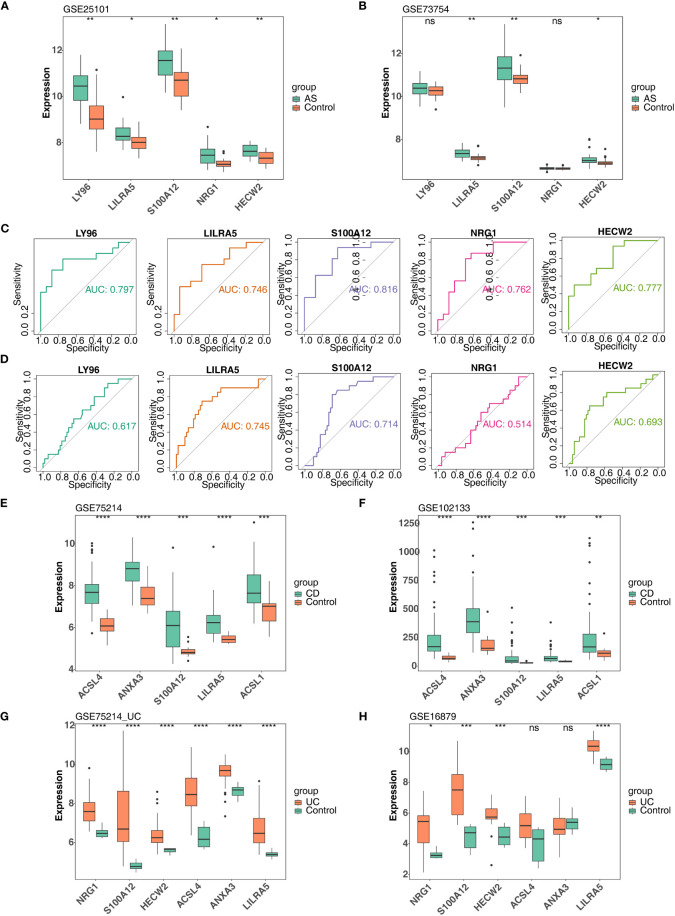
Verification of feature genes between control and disease (AS, CD and UC) groups. **(A, B)** 5 AS-candidate-feature genes expression levels between AS and control groups (training & validation sets); **(C, D)** Plotted ROC curves for 5 AS-candidate-feature genes in AS group (training & validation sets); **(E, F)** 5 CD-candidate-feature genes expression level differences between CD and controls (training & validation sets); **(G, H)** 5 UC-candidate feature genes expression level differences between UC and controls (training & validation sets). ns, no significance; *, **, ***, and **** indicate the significance of gene expression differences, the more asterisks there are, the greater the significance of the difference; *: p < 0.05; **: p < 0.01; ***: p < 0.001; ****: p < 0.0001.

**Figure 7:**
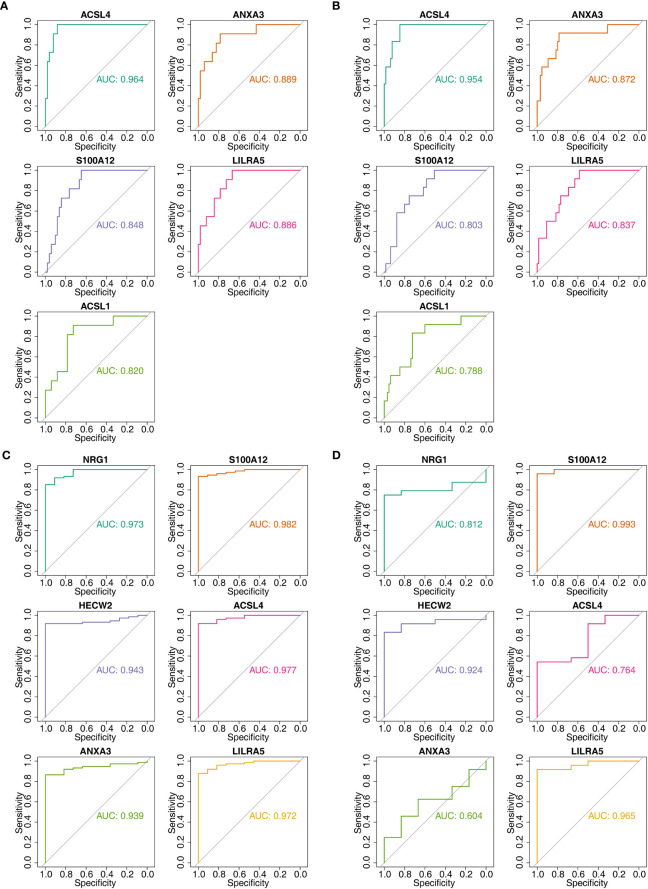
The AUC values of candidate feature genes involved in AS, CD and UC. **(A, B)** Plotted ROC curves for 5 CD-candidate-feature genes in CD groups (training & validation sets); **(C, D)** Plotted ROC curves for 6 UC-candidate-feature genes in UC groups (training & validation sets).

In order to further obtain diagnostically significant feature genes, we performed expression analysis and painted the ROC curves. For the AS, the expression of *LILRA5*, *S100A12*, and *HECW2* was consistent and significantly different in the GSE25101 and GES73754 datasets (**Figures 6A, B**). Combined with the results of the ROC curves, 2 AS-feature genes (*S100A12* and *LILRA5*) were finally obtained (**Figures 6C, D**). Likewise, a total of 5 CD-feature genes (*ACSL4*, *ANXA3*, *S100A12*, *LILRA5* and *ACSL1*) and 4 UC-feature genes (*NRG1*, *S100A12*, *HECW2*, and *LILRA5*) were obtained (**Figures 6E–H**, **7A–D**).

### 3.5 Construction and evaluation of nomogram

**Figure 8:**
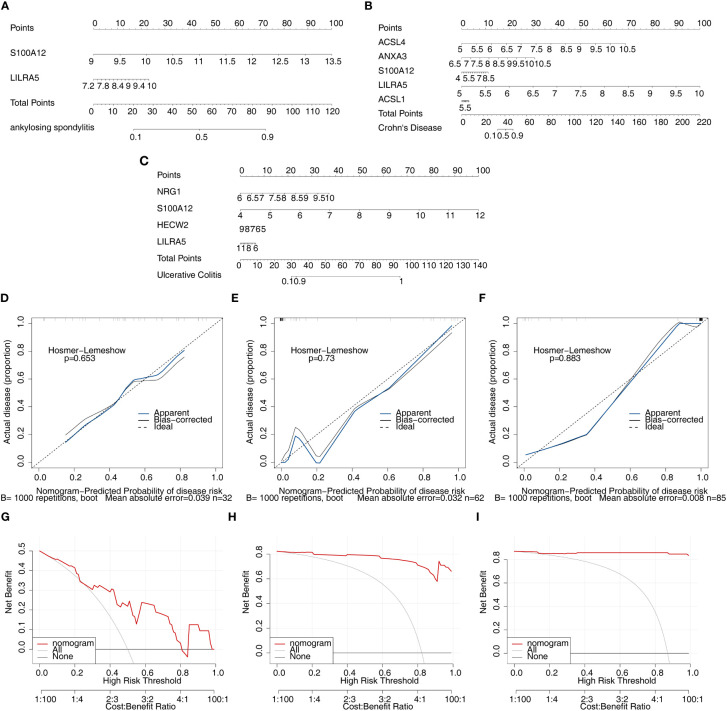
Nomogram model construction and evaluation of candidate feature genes involved in AS, CD and UC. **(A–C)** the nomogram model construction on the basis of feature genes to predict the likelihood of disease in patients with AS, CD and UC (p>0.05; MAE<0.05); **(D–F)** Calibration curve showed the accuracy of the nomogram was relatively high and validated the model performance of AS, CD and UC; **(G–I)** DCA curve suggested net profit of the constructed model is better than the default method.

After screening the feature genes, the nomograms were created to predict the likelihood of disease in patients with three diseases (**Figures 8A–C**). The accuracy of the nomogram was relatively high, which was validated by the calibration curve (**Figures 8D–F**). The results of the DCA curve suggested higher returns for the models (**Figures 8G–I**).

### 3.6 GSEA analysis of co-feature genes

**Figure 9:**
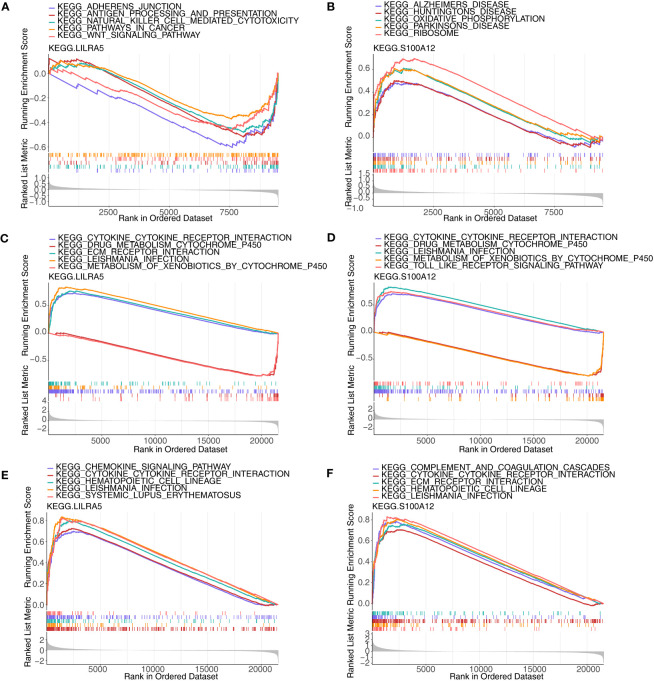
GSEA analysis of each co-feature genes. **(A, B)** Single-gene GSEA of *S100A12* and *LILRA5* on pathways in AS; **(C, D)** Single-gene GSEA of *S100A12* and *LILRA5* on pathways in CD; **(E, F)** Single-gene GSEA of *S100A12* and *LILRA5* on pathways in UC.

Single-gene GSEA was implemented to explore the enriched regulatory pathways and molecular functions of each co-feature genes. Firstly, two feature genes (*S100A12* and *LILRA5*) were obtained. In AS, *LILRA5* low-expression group was mainly enriched to KEGG terms such as adherens junction, antigen processing and presentation, etc. (**Figure 9A**; **Supplementary Table 7**); *S100A12* high-expression group was primarily enriched to KEGG terms such as oxidative phosphorylation, ribosome, etc. (**Figure 9B**). In CD, *LILRA5* and *S100A12* high-expression group were primarily enriched to KEGG pathways, including cytokine-cytokine receptor interaction, etc., the low expression groups were primarily enriched for drug metabolism cytochrome P450 etc. (**Figures 9C, D**; **Supplementary Table 8**). In UC, *LILRA5* and *S100A12* high-expression group were primarily enriched to KEGG pathways, including cytokine-cytokine receptor interaction, hematopoietic cell lineage, etc. (**Figures 9E, F**; **Supplementary Table 9**).

### 3.7 Immune-related analyses of co-feature genes

**Figure 10:**
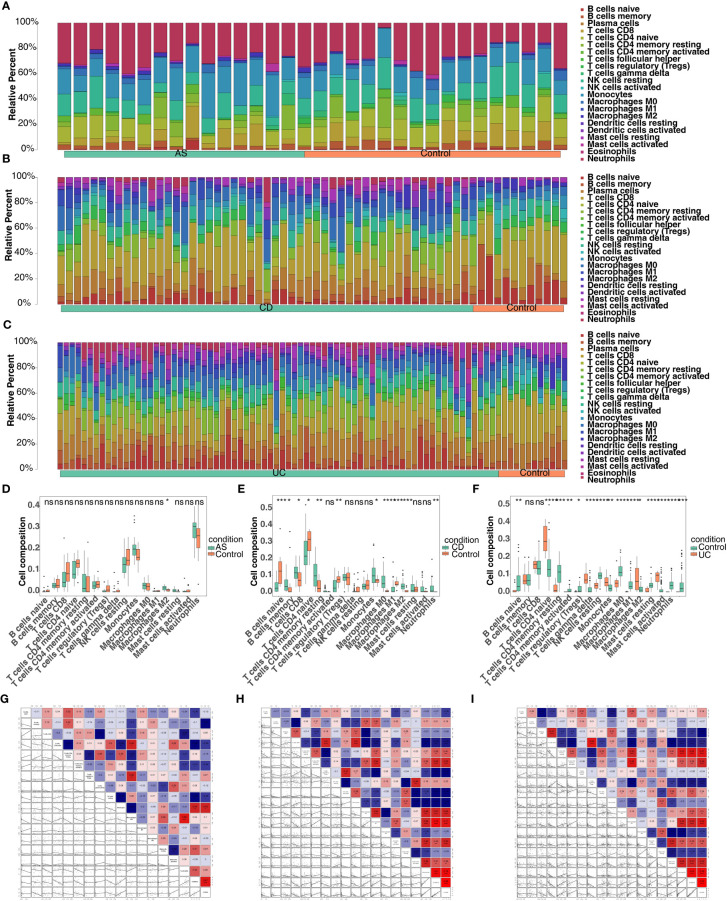
Screening for co-feature genes and immune-infiltration analysis. **(A–C)** The proportion of the 22 immune cells in each sample in AS, CD and UC; **(D–F)** The difference in the proportion of immune cells infiltrating between the three diseases and control group (Wilcoxon method); ns, no significance; *, **, ***, and **** indicate the significance of gene expression differences, the more asterisks there are, the greater the significance of the difference; *: p < 0.05; **: p < 0.01; ***: p < 0.001; ****: p < 0.0001. **(G–I)** The correlation analysis between the proportion of immune cells infiltrating and co-feature genes in AS, CD and UC.

Through the results of single-gene GSEA we found that the co-feature genes were related to immunity, and therefore immune infiltration analysis was carried out. The bars showed the proportion of the 22 immune cells in each sample in the three diseases (**Figures 10A–C**). In total, one immune cell (M2 macrophages) was significantly different between the AS and control groups (**Figure 10D**); Significant differences were observed in the levels of 11 immune cell types (plasma cells, resting memory CD4 T cell, M0 macrophages, neutrophils, etc.), when comparing the CD and control groups (**Figure 10E**); Significant differences were observed in the levels of 15 immune cells (effector memory CD8 T cell, gamma delta T cell, neutrophils, etc.), when comparing the UC and control groups (**Figure 10F**). The correlation analysis indicated that *S100A12* was strongly negatively association with naive CD4 T cells, while it was strongly positively correlated with monocytes in AS (**Figure 10G**); *S100A12* was strongly negatively correlated with CD8 T cell, while it was strongly positively relation with M1 macrophages in CD (**Figure 10H**); *LILRA5* was strongly negatively correlated with CD8 T cell, while it was strongly positively associated with neutrophils (**Figure 10I**).

### 3.8 The TF-mRNA-miRNA network of co-feature genes

**Figure 11:**
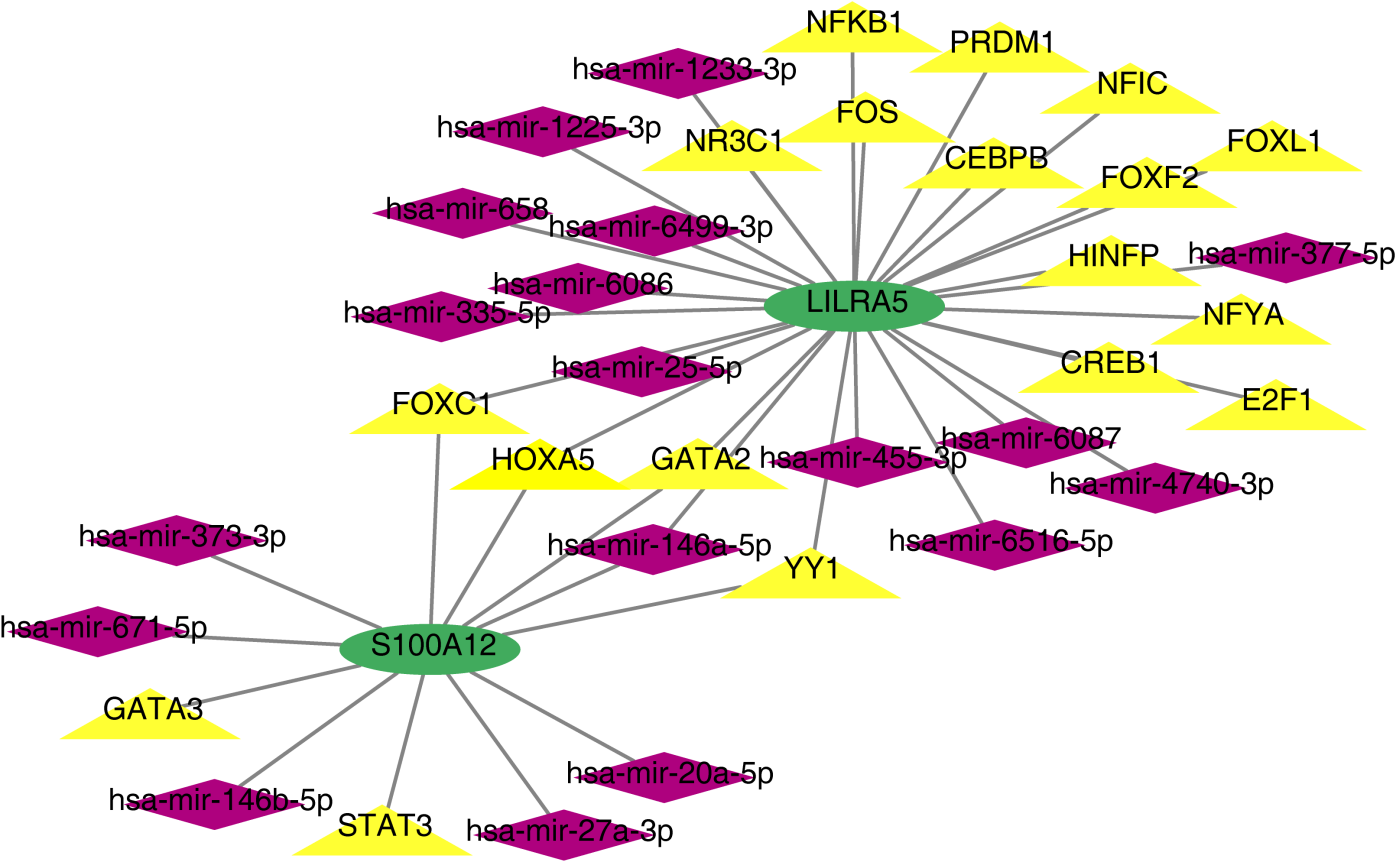
TF-mRNA-miRNA network of *S100A12* and *LILRA5*.

Based on co-feature genes, we obtained 18 miRNAs (hsa-mir-1225-3p, hsa-mir-1233-3p, hsa-mir-4740-3p, hsa-mir-6086 and so on) and 18 TFs (NFKB1, CEBPB, HINFP, CREB1, YY1, etc.). A total of 38 nodes (2 co-feature, 18 miRNAs and 18 TFs) and 41 edges were included in the TF-mRNA-miRNA network (**Figure 11**). Among them, *LILRA5* obtained more miRNAs and TFs.

### 3.9 The expression levels of *S100A12* and *LILRA5*

**Figure 12:**
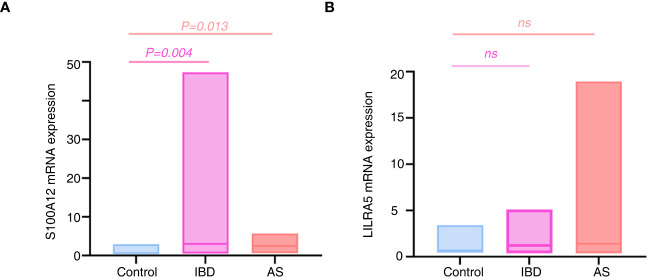
The mRNA expression differences of *S100A12* **(A)** and *LILRA5* **(B)** between healthy controls and diseases. *S100A12* mRNA expression was significantly up regulated in the IBD and AS patients compared with healthy individuals. ns, no significance.

Because of the significant associations of *S100A12* and *LILRA5* with AS and IBD, the mRNA expression levels of the two genes in PBMCs extracted from 34 participants (12 healthy controls, 8 AS patients, 12 UC patients and 2 CD patients) were tested. Significant associations were shown between *S100A12* mRNA expression with AS and IBD (P < 0.05, **Figure 12A**; **Supplementary Table 10**). No correlation was detected between *LILRA5* mRNA expression with AS and IBD (P > 0.05, **Figure 12B**).

### 3.10 Causal association between *S100A12* and AS/UC/CD

**Figure 13:**
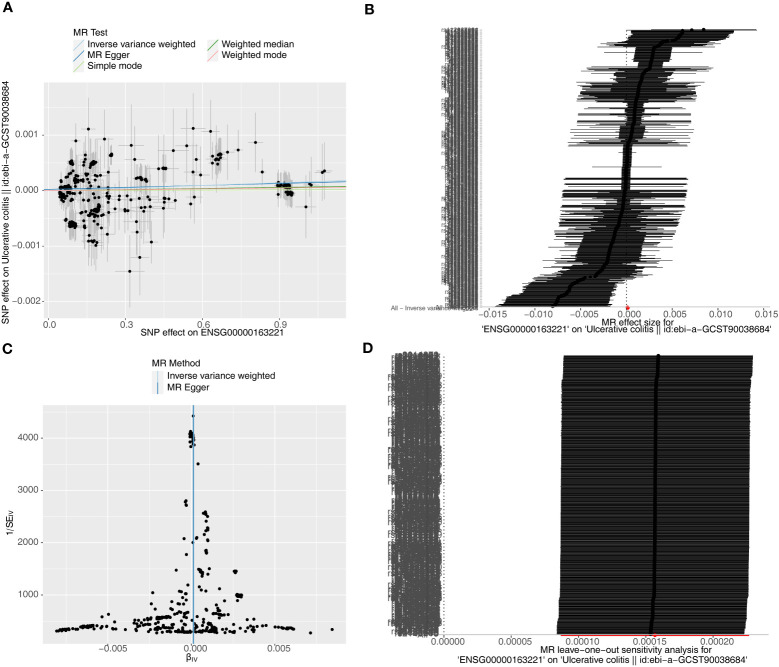
The significant outcomes of MR effect regarding *S100A12* on UC. **(A)** Scatterplot: The x-axis represents the effect of SNPs on exposure, and the y-axis represents the effect of SNPs on the outcome. A slope greater than 0 indicates that the exposure factor is an adverse factor for the outcome. **(B)** Forest plot: A value greater than 0 implies a positive association between the SNP position and the outcome, while a value less than 0 suggests a negative association. **(C)** Funnel plot. **(D)** Leave-one-out: Leave-one-out analysis did not result in the exclusion of any instrumental variable, and the model’s effects remained statistically significant without significant deviations.

**Figure 14:**
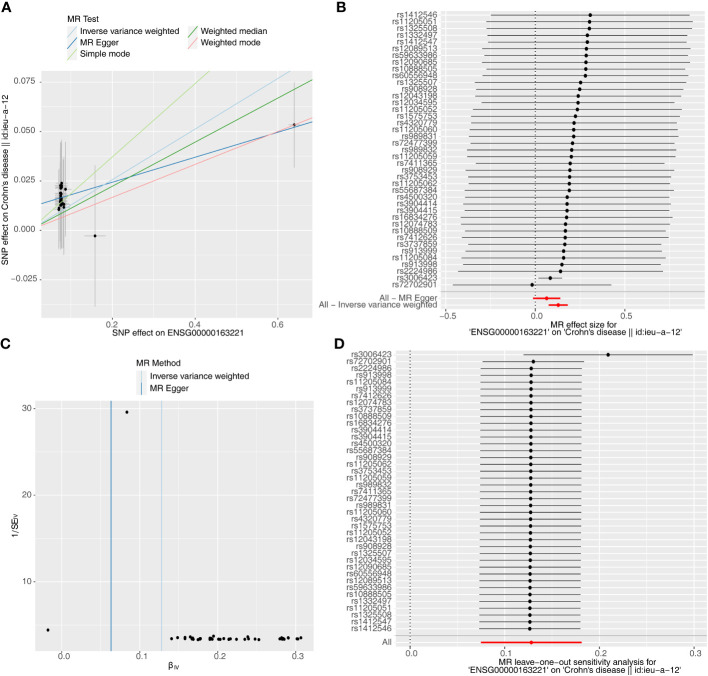
The significant outcomes of MR effect regarding *S100A12* on CD. **(A)** Scatterplot: The x-axis represents the effect of SNPs on exposure, and the y-axis represents the effect of SNPs on the outcome. A slope greater than 0 indicates that the exposure factor is an adverse factor for the outcome. **(B)** Forest plot: A value greater than 0 implies a positive association between the SNP position and the outcome, while a value less than 0 suggests a negative association. **(C)** Funnel plot. **(D)** Leave-one-out: Leave-one-out analysis did not result in the exclusion of any instrumental variable, and the model’s effects remained statistically significant without significant deviations.

Causal relationship between *S100A12* and the diseases (AS, UC and CD) was further explored in the study. Using the IVW method, we found a significant correlation between the *S100A12* gene and the risk of developing UC and CD, with an OR of 1.000157 (95% confidence interval = 1.000087-1.000227, p = 1.10E-05) in UC and an odds ratio (OR) of 1.003 (95% confidence interval = 1.000-1.005, p = 0.006) in CD (See **Supplementary Table 1**, **Figures 13A, B**, **14A, B**). The funnel plot of causal effects appeared approximately symmetrical (**Figures 13C**, **14C**) and the intercept of the MR Egger regression did not indicate horizontal pleiotropy, further confirming the absence of bias in the causal effect. As shown in **Figures 13D** and **14D**, we conducted systematic MR analysis on the remaining SNPs after removing each SNP. The results remained consistent, indicating that the causal relationships were significant for all SNPs. This also suggests that there are no dominant SNPs at the *S100A12* gene level associated with UC and CD, validating the previous MR results (**Supplementary Tables 11**, **12**).

## 4 Discussion

The association between AS and IBD has been extensively documented. The coexistence of both conditions can result in severe symptoms and unfavorable outcomes, often leading to misdiagnosis and inadequate treatment (8). Recently identified as a novel form of cell death, disulfidptosis is triggered by the accumulation of excessive cysteine within cells, leading to disulfide stress (20). This phenomenon is typically triggered during glucose starvation, potentially contributing to the organism’s internal environmental equilibrium (30). Considering its discovery, we have undertaken this study to explore its potential association with both diseases and its potential role in influencing the co-occurrence of these two conditions. After analyzing both the training and validation sets of the three diseases, we identified the following feature genes: 2 AS-feature genes (S100A12 and LILRA5); 5 CD-feature genes (ACSL4, ANXA3, S100A12, LILRA5, and ACSL1); and 4 UC-feature genes (NRG1, S100A12, HECW2, and LILRA5). Subsequently, a total of two co-feature disulfidptosis-related genes, namely *S100A12* and *LILRA5*, were obtained. Further functional experiments validated the significant correlation between *S100A12* mRNA expression levels with AS and IBD. In addition, by MR analysis, we identified a causal relationship of *S100A12* in the development of IBD (UC and CD).

The long-chain acyl-CoA synthetase family (ACSL), located on the outer mitochondrial membrane and endoplasmic reticulum, catalyzes the conversion of fatty acids to acyl-CoA. Serving as intermediates in the lipid metabolic pathway, acyl-CoAs participate in various biological processes, including the maintenance of cell membrane structure, energy metabolism, and lipid metabolism (31). Among the key subtypes, ACSL1 and ACSL4 have been identified as crucial players. ACSL1 was recently found to be a promoter of iron accumulation, while ACSL4 was considered instrumental in integrating polyunsaturated fatty acids (PUFA) into phospholipids, a significant event in iron accumulation (31, 32). Iron accumulation was a form of non-apoptotic cell death driven by lipid peroxidation, with lipid metabolism being a major metabolic change during the process. In patients with IBD, encompassing CD and UC, the inflammatory process led to damage of the intestinal mucosa and ulcer formation. This inflammatory state might potentially result in local iron accumulation, initiating oxidative stress and activating the iron accumulation pathway (33). Although the specific mechanisms by which *ACSL1* and *ACSL4* participate in and regulate iron accumulation have not been fully understood, several studies suggested that they may serve as therapeutic targets for inhibiting iron accumulation (31, 32). While the relationships of these genes with disulfidptosis have not been extensively studied, the aforementioned researches have suggested their potential associations with other cell death pathways. Through bioinformatics approaches, we have, for the first time, confirmed their relevance to the disulfidptosis process in CD, but further research and validation are needed to reveal the molecular mechanisms at play.

Neuregulin 1 (NRG1) was initially identified as a 44-kD glycoprotein that interacted with the NEU/ERBB2 receptor tyrosine kinase, enhancing phosphorylation on its tyrosine residues (34). The interaction between *NRG1* and *ErbB4* is believed to play a role in the pathological mechanisms of schizophrenia. Positive outcomes for anxiety disorders and schizophrenia patients have been suggested through targeted interventions affecting mutations in *NRG1* and *ERBB4* (35). Additionally, under stress conditions, including viral infection, cytotoxic agents, and oxidative stress, the activation of *NRG-1/ERBB* signaling has been shown to protect myocardial cells from apoptosis. Although research on intestinal diseases is limited, genetic variations located at the *NRG1* have been found to increase the risk of congenital megacolon. Annexin A3 *(ANAX3)* is a member of the annexin family, a calcium-dependent phospholipid-binding protein family that plays a role in regulating cell growth and signaling pathways (36). The function of this protein is to inhibit A2 phospholipase and cleave inositol 1,2-cyclic phosphate to form inositol 1-phosphate (37). Recent studies showed that the ANXA3-specific expression was significantly higher in AS patients than in normal controls, with a significant statistical difference. Moreover, ANXA3 was found positively correlated with neutrophils, and the expression of neutrophils in AS patients was significantly higher than in the control group (38). While the research on NRG1 and ANAX3 in autoimmune diseases is limited, our findings have identified them as IBD-feature genes linked to disulfidptosis. Recognizing the intricate association between apoptosis, neutrophils, and the onset of IBD, we believe further investigation into the mechanisms is necessary.

Leukocyte Immunoglobulin-Like Receptor Subfamily A Member 5 (*LILRA5*) is a gene that encodes a protein belonging to the immunoglobulin superfamily (39). It is a type I transmembrane receptor that is expressed on various immune cells, including macrophages, monocytes and dendritic cells. Stimulating this receptor on the surface of monocytes has been demonstrated to induce calcium flux and the secretion of several proinflammatory cytokines. This suggests that this protein plays a role in initiating innate immune responses (40). In a study involving RA patients, *LILRA5* was found to be linked with the Immunoreceptor Tyrosine-based Activation Motif (ITAM) of the Fc receptor common γ chain (39, 41). Activation of *LILRA5* on monocyte surfaces led to elevated phosphorylation of tyrosine kinases, which in turn led to the early and specific production of pro-inflammatory cytokines such as TNF-α, IL-6 and IL-1β, followed by a delayed induction of IL-10. Taken together, these findings imply that *LILRA5* could potentially play a role in the onset of RA (39). The expression of *LILRA5* mRNA and protein was observed to be notably affected by both macrophage differentiation and the *in vitro* treatment of monocytes with cytokines (TNF-α, IFN-γ and IL-10). This indicates that the activation of the *LILRA5* receptor is tightly controlled by cytokines that are produced when it is stimulated. In our study, *LILRA5* was identified as a diagnostic co-feature gene of AS, CD and UC, and although the RT-PCR results failed to detect an association between *LILRA5* mRNA and IBD and AS, aforementioned research allowed us to speculate on the potential mechanisms of *LILRA5* in these two conditions. Considering the well-established roles of IFN-γ, IL-10, and TNF-α in the pathogenesis of AS, CD and UC (42), *LILRA5* may accelerate the development of these conditions due to the activation of cytokine pathways. It is essential to conduct further investigations with larger sample sizes to explore this relationship more thoroughly.

*S100A12* is an alarm signal, selectively targeting granulocytes and binding to RAGE and TLR4 receptors (43). Research suggests that the activation of NF-κB through RAGE dependency can lead to the secretion of pro-inflammatory cytokines, ultimately culminating in the recruitment of monocytes (44, 45). In mouse models, *S100A12* has been shown to play a role in the recruitment of inflammatory cells. Furthermore, it has been observed to be over-expressed in inflamed tissues of individuals with various conditions, such as IBD, Psoriatic arthropathies (PsA), Juvenile Idiopathic Arthritis (JIA), and Rheumatoid Arthritis (RA) (46–48). Notably, *S100A12* is a dependable biomarker for both IBD and systemic-onset JIA (49). As JIA and AS are believed to be autoimmune disorders the immune system mistakenly attacks the body’s own tissues, leading to inflammation and harm to the joints, it suggests that *S100A12* might have a significant role in the development of AS and IBD. Our study validated *S100A12* as a diagnostically co-feature gene of AS, CD and UC, and the significant associations were also validated between *S100A12* mRNA expression and diseases. In both AS and IBD patients, there was a significant upregulation of *S100A12* mRNA expression compared to healthy individuals. We proceeded with MR analysis, uncovering a causal link between *S100A12* and the onset of UC and CD. In line with the gene transcription findings, it becomes evident that the upregulation of *S100A12* significantly contributes to the pathogenesis of both UC and CD.

Single-gene GSEA was employed to investigate the enriched regulatory pathways and molecular functions of *S100A12* and *LILRA5*. In AS, the *LILRA5* low-expression group was primarily enriched to adherens junction, antigen processing and presentation, while *S100A12* exhibited high levels of enrichment in oxidative phosphorylation. Among them, aberrant antigen processing and presentation has been recognized as key pathogenic factors leading to immune activation in AS (50). Recent research indicated that oxidative phosphorylation might be considered as a common pathogenic factor for both AS and dementia (51). The adherens junction plays a crucial role in establishing physical connections between cells and governing cell-cell contacts, which are essential for the morphogenesis and remodeling of tissues and organs (52). There is a lack of documented research investigating the connection between adherens junctions and AS. However, abnormal cadherin expression in immune cells can lead to modified interactions among immune cells, contributing to the onset of autoimmune diseases (53). Our research showed that *LILRA5* and *S100A12* high-expression groups were mainly enriched to cytokine-cytokine receptor interaction both in CD and UC. The engagement between cytokines and their respective receptors initiates downstream signaling pathways, resulting in the release of pro-inflammatory molecules and the recruitment of immune cells to the inflamed intestinal tissues (54, 55). This persistent inflammation can lead to tissue damage, disrupting the integrity of the gut barrier and contributing to the development of IBD, including CD and UC (56).

Immune-related analyses of co-feature genes were also carried out in our study. In AS, there was a significant negative correlation between *S100A12* and naive CD4 T cells, while a strong positive correlation was observed between *S100A12* and monocytes. Studies have shown that individuals with AS may exhibit increased activation of monocytes (57). Monocytes in individuals with AS may have an altered cytokine production profile, favoring the release of pro-inflammatory cytokines like TNF-α and IL-1β, which are known to be key drivers of inflammation in AS (58). The decrease in naive CD4 T cells has shown to be related to an increase in the differentiation of these cells into various effector T cell subsets, such as Th1 and Th17 cells. Studies have suggested an increase in pro-inflammatory Th1 and Th17 CD4 T cell proportions in individuals with AS (59). In CD group, there was a negative correlation between *S100A12* and CD8 T cells, whereas a strong positive correlation was observed between S100A12 and M1 macrophages. There has been evidence of increased activation of M1 macrophages in the inflamed intestinal tissues of CD patients (60). In individuals with IBD, there is evidence of increased activation of CD8 T cells in the intestinal mucosa. These activated CD8 T cells can produce pro-inflammatory cytokines and chemokines, contributing to the inflammation of the disease (61). Furthermore, *LILRA5* was strongly negatively correlated with CD8 T cell, while it was strongly positively associated with neutrophils in UC group. Neutrophils have long been acknowledged as crucial components of the immune system, contributing to both innate and adaptive immunity. Recent studies have uncovered significant phenotypic and functional irregularities in neutrophils across various systemic autoimmune disorders (62).

In the present study, the feature genes associated with disulfidptosis shared among AS, CD and UC were uncovered, and machine learning and functional enrichment analysis were utilized to identified co-feature genes. To make the results more comprehensive and reliable, we therefore performed functional validation experiments and confirmed the significant role of *S100A12* in AS and IBD. Additionally, by MR analysis, we identified a significant causal relationship between *S100A12* and IBD. There are still several limitations in this study. Firstly, because of the inclusion of multiple diseases and the relative rarity of patients in the active disease phase, we could only to collect a limited number of patient samples for functional studies, therefore, it is crucial to conduct further research with a larger sample size. Secondly, as disulfidptosis is a recent research advancement, additional mechanistic studies are necessary to confirm its relevance to the three conditions, constructing mouse or cell models for functional experiments could further validate the reliability and accuracy of our results. Thirdly, the RT-PCR data for LILRA5 did not exhibit significant correlation, potentially influenced by factors such as sample size, qRT-PCR reaction conditions, and tissue heterogeneity. Considering the mentioned drawbacks, we aim to enhance this study by conducting more comprehensive mechanistic investigations in the future.

## 5 Conclusions

Our study, for the first time, identified two co-feature genes (*S100A12* and *LILRA5*) associated with disulfidptosis in AS, CD, and UC. Investigating the functions of these genes and pathways in modulating autoimmunity holds promise for future therapeutic breakthroughs in the management of AS, CD, and UC.

## Data availability statement

The datasets presented in this study can be found in online repositories. The names of the repository/repositories and accession number(s) can be found in the article/**Supplementary Material**.

## Ethics statement

The studies involving humans were approved by the First Affiliated Hospital of Zhengzhou University (2021-KY-0246-001) and the First Affiliated Hospital of Chongqing Medical University (No. 2009-201008). The studies were conducted in accordance with the local legislation and institutional requirements. The participants provided their written informed consent to participate in this study.

## Author contributions

LL: Conceptualization, Data curation, Formal analysis, Validation, Writing – original draft, Writing – review & editing. HF: Data curation, Methodology, Validation, Writing – original draft, Writing – review & editing. FL: Data curation, Formal analysis, Methodology, Validation, Writing – review & editing. KX: Data curation, Formal analysis, Validation, Writing – review & editing. PZ: Validation, Writing – review & editing. HZ: Validation, Writing – review & editing. XJ: Validation, Writing – review & editing. RS: Formal analysis, Funding acquisition, Supervision, Validation, Writing – original draft, Writing – review & editing. PY: Formal analysis, Funding acquisition, Methodology, Supervision, Validation, Writing – original draft, Writing – review & editing. DL: Data curation, Formal analysis, Funding acquisition, Methodology, Supervision, Validation, Writing – original draft, Writing – review & editing.

## Supplementary material

Supplementary Data 1 3 datasets related to the diseases from the GEO database (https://www.ncbi.nlm.nih.gov/geo/).

Supplementary Data 2 3 datasets related to the diseases from the GEO database (https://www.ncbi.nlm.nih.gov/geo/).

Supplementary Data 3 The code involved in the study.
